# Dynamics of Vaginal and Rectal Microbiota Over Several Menstrual Cycles in Female Cynomolgus Macaques

**DOI:** 10.3389/fcimb.2019.00188

**Published:** 2019-06-12

**Authors:** Marie-Thérèse Nugeyre, Nicolas Tchitchek, Cindy Adapen, Claude Cannou, Vanessa Contreras, Fahd Benjelloun, Jacques Ravel, Roger Le Grand, Romain Marlin, Elisabeth Menu

**Affiliations:** ^1^CEA, Université Paris-Sud, Inserm, U1184 “Immunology of Viral Infections and Autoimmune Diseases” (IMVA), IDMIT Department, IBFJ, Fontenay-aux-Roses, France; ^2^MISTIC Group, Department of Virology, Institut Pasteur, Paris, France; ^3^Institute for Genome Sciences and Department of Microbiology and Immunology, University of Maryland School of Medicine, Baltimore, MD, United States

**Keywords:** cynomolgus macaques, female hormones, microbiota, mucosa, rectum, vagina

## Abstract

The composition of the microbiota in cynomolgus macaques is only partially characterized, although this animal model is often used to study pathogenesis and preventive strategies against infections. We thus performed, for the first time, a longitudinal characterization of the vaginal and rectal microbiota of five cycling female cynomolgus macaques. Samples were collected weekly for 15 weeks and the V3/V4 regions of the16S rRNA gene sequenced. Sequences were analyzed with QIIME for OTU detection and taxonomic assignment. Progesterone levels were also determined to evaluate hormonal influence on bacteria relative abundance. The rectal and vaginal bacterial composition in cynomolgus macaques is polymicrobial and clearly distinct, with larger individual variability in the vagina. Rectal microbiota profiles were consistent between animals, whereas they were highly variable and animal-specific in the vagina. In the rectum, the most abundant taxa were *Ruminococcaceae, Prevotella*, and *Clostridiales*. In the vagina, the most abundant genera were *Sneathia, Porphyromonas, Prevotella*, and *Fusobacterium*. *Lactobacillus* were found at relative abundances higher than 1% in only one animal and were not predominant. Comparison of the vaginal cynomolgus macaque microbiota with that of humans showed similarity to community state type IV-A usually associated with dysbiosis. In the vagina, the relative abundance of 12 bacterial genera was found to be associated with progesterone levels. Our study provides a detailed characterization of the rectal and vaginal microbiota in female cynomolgus macaques and opens new perspectives of this animal model.

## Introduction

Mucosae are the main portal of entry of sexually transmitted pathogens. Several factors of the mucosal environment are known to maintain the integrity of the epithelial barrier and protect against infections (Ferreira et al., 2014). Studies of mucosae-associated microbiota are increasing exponentially and have demonstrated their impact on the protection against sexually transmitted infections (STI). It has been shown that the microbiota can influence mucosal and systemic immune functions (Manuzak et al., 2016), as well as host physiology and in pathological states (Clemente et al., 2012; Anahtar et al., 2015).

The human gut microbiota is composed primarily of four major bacterial phyla: *Firmicutes, Bacteroidetes, Actinobacteria*, and *Proteobacteria*. It is involved in the maturation of the host immune system and many basic metabolic pathways, including sugar and protein fermentation (Landman and Quevrain, 2016). Dysbiosis of the gastrointestinal microbiota is associated with HIV-1 disease progression (Vujkovic-Cvijin et al., 2013).

The human vaginal microbiota plays an important role in the maintenance of an environment that protects against viral or bacterial infections, including HIV-1 (Spear et al., 2011). *Lactobacillus* spp. constitute the most common bacterial genus in women (Giorgi et al., 1987; Eschenbach et al., 1989; Antonio et al., 1999; Ravel et al., 2010). Five community state types (CST) have been defined in women: CST I (*Lactobacillus crispatus* predominantly), CST II (*Lactobacillus gasseri* predominantly), CST III (*Lactobacillus iners* predominantly), CST IV (comprises of a wide array of strict and facultative anaerobes), and CST V (*Lactobacillus jensenii* predominantly). CST IV has been further divided in CST IV-A and IV-B according to the modest proportions of *Lactobacillus* spp. (CST IV-A) and the different proportions of various species of strictly anaerobic bacteria in the sub-classes (Gajer et al., 2012). *Lactobacillus* spp. can maintain a protective environment against STI by producing several factors, including lactic acid, which decreases the pH (~4), and bacteriocins (Petrova et al., 2013; Tachedjian et al., 2018). The composition of the vaginal microbiota varies over time depending on estrogen levels over a woman's lifespan (Cribby et al., 2008). Vaginal dysbiosis is characterized by a diverse microbiota with a greater abundance of anaerobic bacteria, impaired epithelial integrity, and enhanced microbial translocation (Ziklo et al., 2016). It also induces the production of pro-inflammatory cytokines (such as TNF-α or IL-8) (Anahtar et al., 2015; Borgdorff et al., 2016a), decreases mucin expression (Borgdorff et al., 2016b), and stimulates the activation of CCR5^+^ CD4^+^ T cells (Anahtar et al., 2015). Dysbiosis can lead to bacterial vaginosis (BV), the most common vaginal condition of women in reproductive age. BV can be symptomatic or asymptomatic. In women with BV, there are reduced proportions of *Lactobacillus* spp. and increases in the number and diversity of facultative and strictly anaerobic bacteria, including species of *Gardnerella, Prevotella*, and other taxa of the order *Clostridiales* (Fredricks et al., 2005). Vaginal and gastrointestinal live biotherapeutic products have been proposed to prevent and cure dysbiosis, and restore a functional microbiota (Ganesh and Versalovic, 2015). These include *Lactobacillus* spp. formulated as live biotherapeutic products to prevent and treat bacterial vaginosis (BV) (Homayouni et al., 2014; Braundmeier et al., 2015).

Macaques are relevant models to study pathogenesis and validate preventive strategies against the transmission of infections (Alfson et al., 2017; Sharpe et al., 2017). The menstrual cycle length of cynomolgus macaques (28–32 days) is similar to that of humans (28–30 days) and do not exhibit seasoning menstrual cycle in contrast to rhesus macaques (Weinbauer et al., 2008). Currently, the vaginal microbiota of rhesus (Spear et al., 2010) and pigtailed macaques (Spear et al., 2012) have been described, but not that of cynomolgus macaques. Additionally, the fecal microbiota has been described in cynomolgus macaques (Seekatz et al., 2013) of different origins (Mauritius, Indonesia, and the Philippines) and the analysis was performed on samples collected over a short period of time (14 days). More recently, a gut microbiome gene catalog from cynomolgus macaques was reported and compared with pig, mouse, and human gut microbiomes (Li et al., 2018). A metagenomic comparison of the rectal microbiota between rhesus and cynomolgus macaques was also performed (Cui et al., 2018).

The aim of this study was to perform a longitudinal characterization of the vaginal and rectal microbiota of cynomolgus macaques over several menstrual cycles. Sequencing of the 16S rRNA gene was performed on samples collected once a week for 15 weeks, which covers at least three menstrual cycles per animal. This study, characterizing the mucosal bacterial composition of female cynomolgus macaques, further improve our understanding of the relationship between the microbiota and hormonal cycle.

## Materials and Methods

### Animal Housing

Five sexually mature macaques (*Macaca fascicularis*) were imported from Mauritius and housed in the Infectious Disease Models and Innovative Therapies (IDMIT) facilities at the Commissariat à l'Energie Atomique et aux Energies Alternatives (CEA, Fontenay-aux-Roses, France). The animals were housed in groups under controlled conditions of humidity, temperature, and light (12-h light/dark cycles). Water was available *ad libitum*. The animals were monitored and fed with commercial monkey chow (6020 formula, Altromin, Germany) and fruit once or twice a day by trained personnel and were provided with environmental enrichment, including toys, novel foodstuffs, and music, under the supervision of the CEA Animal Welfare Officer. The 6020 formula is a cereal-based (soy, wheat, and corn) fixed formula which is free of alfalfa and fish/animal meal and deficient in nitrosamines. This maintenance diet was designed as complete feeding stuff for adult NHP. The five animals in this study were housed in two different rooms into level-3 facilities. They were between 3 and 5 years old, weighed between 3.09 and 3.79 Kg, had different MHC genotypes (Table 1), and were never pregnant.

**Table 1:** Animal characteristics.

**Animal ID**	**Age (years)**	**MHC genotype**	**Weight (Kg)**	**Room #**
BA890I	4	H5/H3	3.79	1
CA086	5	H2/H1	3.53	2
CB804C	4	H4/rec.H1-H5-H3	3.38	1
CBL015	4	H3/H1	3.43	1
CCA096	3	rec. H6-H1/rec.H2-H6	3.09	1

### Experiment Design and Sample Collection

**Figure 1:**
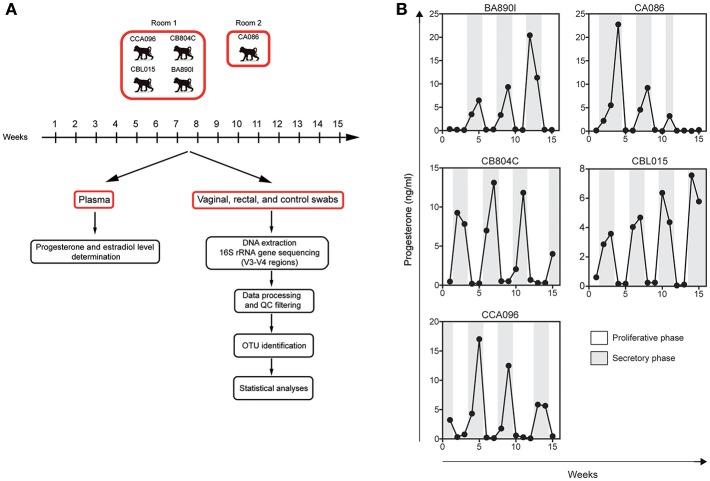
Experimental design and individual progesterone levels. **(A)** Five cynomolgus macaques were included in this study to characterize their vaginal and rectal microbiota. Blood, rectal, and vaginal swabs were sampled weekly during a period of 15 weeks. Four animals were housed in the same room and one animal in another. Room controls (air swab) were performed each week to check for possible contamination or other technical biases. After DNA extraction from the swab material, the bacterial 16S rRNA gene was sequenced using primers targeting the V3 and V4 regions. Sequenced reads were preprocessed and analyzed with QIIME for OTU detection and taxonomic assignment. Statistical analyses were then performed to identify relevant taxa. **(B)** Progesterone levels (black line) were determined in the plasma of the animals once a week for 15 weeks.

Sample collection was performed once a week for 15 weeks (Figure 1A), corresponding to approximately three menstrual cycles. Vaginal and rectal samples were collected with nylon flocked swabs which were stored frozen at −20°C in 1 ml Amies transport medium (ESWABR1, Copan Diagnostics Inc., Murrieta, CA, USA) until DNA extraction. Room control samples (air swab) were collected in parallel with vaginal and rectal samples at each timepoint. Sequencing of the V3/V4 regions of the bacterial 16S rRNA gene was followed by bioinformatics analysis, consisting of data processing, read quality-control filtering (QC), OTU identification, and statistical analyses. The animals were anesthetized using ketamine (10 mg/kg) administered intramuscularly once a week before sampling. Blood samples were also collected weekly and plasma stored at −80°C. At least three progesterone peaks were detected per animal during the study, on average one peak every 3–4 weeks, confirming that the five female macaques had regular hormonal cycles.

### Determination of Progesterone and Estradiol Concentrations

Plasma levels of progesterone (Figure 1B) were determined weekly for the five animals (15 samples per animal) using an ELISA kit from IBL International (Hamburg, Germany). Plasma levels of estradiol were determined at the same timepoints using the Invitrogen^TM^ Novex^TM^ Estradiol Human Elisa kit (Göteborg, Sweden).

### DNA Extraction and 16S rRNA Gene Sequencing

The PowerFecal DNA isolation kit from MOBIO (Qiagen, Courtaboeuf, France) was used following the instructions of the manufacturer. PCR was performed using the 16S rRNA gene Amplicon PCR Forward Primer, 5′ TCGTCGGCAGCGTCAGATGTGTATAAGAGACAGCCTACGGGNGGCWGCAG and the 16S rRNA gene Amplicon PCR Reverse Primer, 5′ GTCTCGTGGGCTCGGAGATGTGTATAAGAGACAGGACTACHVGGGTATCTAATCC, which target the 16S rRNA gene V3 and V4 regions selected from Klindworth et al. publication (Klindworth et al., 2013). PCR was performed using KAPA HIFi HotStart ReadyMix (KAPA Biosystems, Roche, Boulogne Billancourt, France). The following conditions were used: initial denaturation at 95°C for 3 min, followed by 25 cycles consisting of denaturation (95°C for 30 s), annealing (55°C for 30 s), and extension (72°C for 30 s) and a final extension step at 72°C for 5 min. PCR products were purified with AMPure XP beads (Beckman Coulter, Villepinte, France). To prepare DNA libraries for Illumina MiSeq using Nextera XT Index Kit (Illumina), PCR reactions were performed with the primers provided in the kit. The following conditions were used: initial denaturation at 95°C for 3 min, followed by eight cycles consisting of denaturation (95°C for 30 s), annealing (55°C for 30 s), and extension (72°C for 30 s) and a final extension step at 72°C for 5 min. A second purification with AMPure XP beads was performed. Sequencing of the V3/V4 region of the 16S rRNA gene was performed on the Illumina MiSeq platform of Institut Pasteur (Paris, France) following the instructions of “16S Metagenomic Sequencing Library Preparation” (ref: 15044223 Rev.B).

### Sequencing Data Processing and Quality-Control Filtering

Paired-end sequenced reads were assembled using FLASH (Magoc and Salzberg, 2011) with default parameters. Only assembled reads with a length >400 bases were retained for analysis. Sequencing adaptors were removed using cutadapt (Martin, 2011). Reads were trimmed using the FASTX-Toolkit (http://hannonlab.cshl.edu/fastx_toolkit/). Finally, assembled reads with a quality score <28 in more than 95% of the sequence were discarded. The number of assembled and QC-filtered reads per sample ranged from 3,608 to 260,786 for rectal microbial profiles and 1,666–440,707 for vaginal microbial profiles. The number of assembled and QC-filtered reads per sample ranged from 7 to 188 for room control microbial profiles.

### OTU Identification, Taxonomic Assignment, and Statistical Analyses

Microbiota profiles were analyzed using QIIME (version 1.9.1) (Caporaso et al., 2010b). Rectal and vaginal microbiota analyses were performed separately and independently for each animal. OTU picking was performed using the uclust algorithm (Edgar, 2010) with identity set at 97%. Representative sets of sequences were aligned with the PyNAST algorithm (Caporaso et al., 2010a). Taxonomic assignments were performed using the RDP classifier (Wang et al., 2007) trained on the Greengenes database (Desantis et al., 2006). Taxa with abundance <1% in all animals were filtered out and aggregated to provide a complete view of the rectal and vaginal compartments. Alpha diversity was calculated based on the Simpson diversity index. Statistical analyses to identify associations between progesterone level and taxon relative abundances were performed using the MetagenomeSeq's fitZIG algorithm (Paulson et al., 2013). Species-level assignments for *Lactobacillus* associated reads was performed using BLAST (Boratyn et al., 2013). Jensen-Shannon divergences were computed using the philentropy R package.

### Graphical and Multidimensional Scaling Representations

Graphical representations of relative taxonomy abundance were generated using GraphPad Prism version 7 for Windows (GraphPad Software, La Jolla California USA, www.graphpad.com), Tableau Software (version 10, Seattle Washington USA), and R software (https://www.r-project.org/). The tree representation showing the taxa commonly or specifically found in the rectum and vaginal tissues was constructed as the union of the two taxonomic trees generated by QIIME. Multidimensional scaling (MDS) representations were generated based on the SVD-MDS algorithm (Becavin et al., 2011). Distances between microbial profiles were computed as Euclidian distances between their relative abundances of identified taxa. The Kruskal Stress indicated in each MDS representation corresponds to the percentage of information lost in the dimensionality reduction process.

## Results

### Rectal and Vaginal Microbiota Are Highly Diverse With Greater Variability Between Individuals in the Vaginal Compartment

Five cynomolgus macaques were included in this study to characterize their vaginal and rectal microbiota. Sample collection was performed once a week for 15 weeks (Figure 1A), corresponding to approximately three menstrual cycles. Plasma levels of progesterone (Figure 1B) were determined weekly for the five animals.

We first aimed to identify the taxa present in the rectal and vaginal microbiota of the five cynomolgus macaques, regardless of their kinetics, and quantify their relative abundance. We identified taxa present in the rectum and vagina for each animal and then combined OTU having the same taxonomic level for each compartment to account for potential individual variability. Only taxa identified with an abundance >1% in at least one animal and one timepoint were considered.

**Figure 2:**
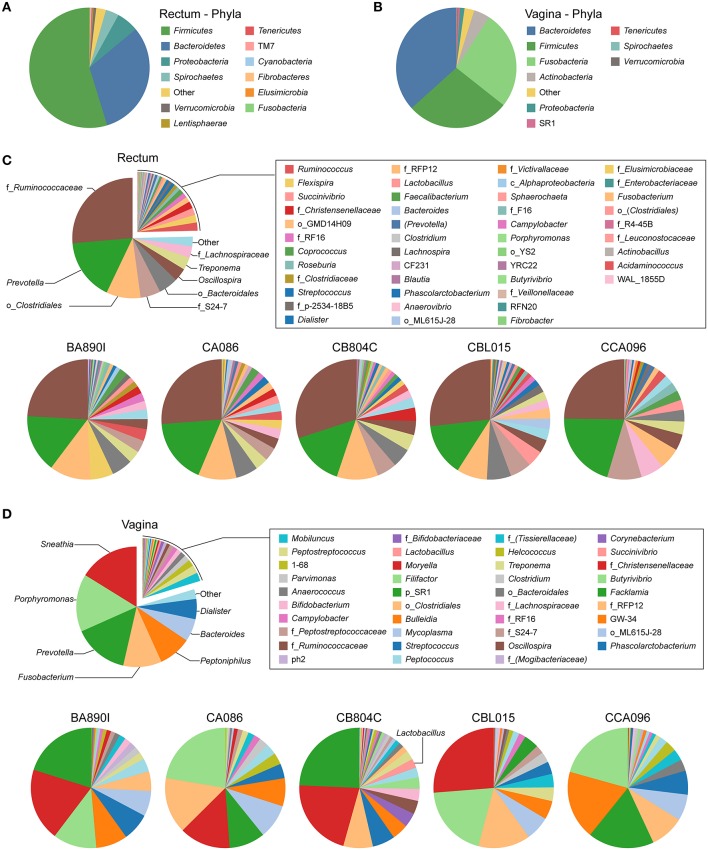
Relative abundance of identified taxa in rectal and vaginal samples. Analyses of relative taxa abundance were performed independently for rectal and vaginal samples. The means of the relative taxa abundances of the five animals are displayed using pie chart representations (**A**,**B** and top panels of **C,D**). The means of the relative taxa abundances for each animal are also represented (bottom panels of **C,D**). The most abundant taxa are indicated on the chart while other taxa are indicated in the legend. For each taxon, the name of the genus is indicated, when possible. In other cases, the family (f_), order (o_), class (c_), or phylum (p_) of the taxa are indicated.

We identified taxa from 12 phyla in rectal samples and nine phyla in vaginal samples (Figures 2A,B). *Firmicutes* was the major phylum detected in the rectum of all the animals, whereas *Bacteroidetes* was the major phylum in the vagina. This was followed by *Bacteroidetes* in the rectum and *Firmicutes* and *Fusobacteria* in the vagina. In the rectum, *Fusobacteria* represented <1% of the bacteria. *Proteobacteria* were detected in both the rectum and vagina at >1% abundance, together with *Spirochaetes* in the rectum and *Actinobacteria* in the vagina.

Individually, the most representative phyla in the rectum were, in order of decreasing abundance: *Firmicutes, Bacteroidetes, Proteobacteria*, and *Spirochaetes*, except for one animal (CB804C) that had a higher abundance of *Spirochaetes* than *Proteobacteria* (Supplementary Table 1). In the vagina, more than 90% of the bacteria were comprised of *Bacteroidetes, Firmicutes*, and *Fusobacteria*, but not always in the same order of abundance, except for animal CB804C (Supplementary Table 2). CB804C vaginal samples had a greater abundance of *Actinobacteria* than the other animals. CBL015 had a higher and CCA096 a lower abundance of *Fusobacteria*. *Actinobacteria* and *Proteobacteria* were also present in all the animals.

We identified 31 genera in cynomolgus macaque rectal samples (Figure 2C and Supplementary Table 3), and when the genus or subsequent taxa could not be assigned, we identified 15 families, six orders, and one class. Among all the animals, seven genera, five families, and three orders had an abundance >1% (Table 2). Thirteen of the 15 identified taxa with an abundance >1% in the entire cynomolgus macaque population were identified in all animals and three (f_*Ruminococcaceae, Prevotella*, and o_*Clostridiales*) were identified in all rectal samples (Table 2).

**Table 2:** Most abundant taxa identified in the macaque rectal microbiota.

		**Present in (%)**^**a**^	
**Taxa**	**% of sequences^**b**^**	**Samples**	**Animals**
f_*Ruminococcaceae*	26.3	100	100
*Prevotella*	16.5	100	100
o_*Clostridiales*	9.2	100	100
f_S24-7	5.4	93	100
o_*Bacteroidales*	5.3	99	100
*Oscillospira*	3.5	99	100
*Treponema*	3.4	96	100
f_*Lachnospiraceae*	3.1	88	100
*Ruminococcus*	2.2	83	100
*Flexispira*	2.1	36	80
*Succinivibrio*	1.9	49	100
f_*Christensenellaceae*	1.9	60	100
o_GMD14H09	1.4	56	80
f_RF16	1.3	51	100
*Coprococcus*	1.3	48	100
Other	2.6	99	100

a Present at ≥1%.

b *Average number of sequences in the 75 samples from the five animals*.

The most abundant taxa in the rectum (Figure 2C, lower pie charts) were, in order of decreasing abundance: f_*Ruminococcaceae* (min 24.1%–max 30.0%)*, Prevotella* (14.1–20.6%), and o_*Clostridiales* (5.8–11.1%), except for one animal (CCA096). For CCA096, f_S24-7 (9.3%) and f_*Lachnospiraceae* (6.2%) were more abundant than o_*Clostridiales* (5.8%).

We identified 32 genera in cynomolgus macaque vaginal samples (Figure 2D and Supplementary Table 4), and when the genus or subsequent taxa could not be assigned, we identified 10 families, three orders, and one phylum. Among all animals, 14 genera and one family had an abundance >1% (Table 3). Eleven of the 15 identified taxa with an abundance >1% in the entire population were identified in all animals, and none were identified in all vaginal samples (Table 3).

**Table 3:** Most abundant taxa identified in the macaque vaginal microbiota.

		**Present in (%)**^**a**^	
**Taxa**	**% of sequences**^**b**^	**Samples**	**Animals**
*Sneathia*	16.2	77	80
*Porphyromonas*	15.5	97	100
*Prevotella*	14.8	87	100
*Fusobacterium*	10.3	83	100
*Peptoniphilus*	8.8	99	100
*Bacteroides*	6.1	61	100
*Dialister*	5.5	95	100
*Mobiluncus*	2.3	69	100
*Peptostreptococcus*	2.2	79	100
1–68	1.8	55	100
*Parvimonas*	1.6	59	80
*Anaerococcus*	1.4	39	100
*Bifidobacterium*	1.2	28	80
*Campylobacter*	1.2	44	100
f_*Peptostreptococcaceae*	1.1	32	80
Other	2.6	96	100

a Present at ≥1%.

b *Average number of sequences in the 75 samples from the five animals*.

The most representative genera in the vagina (Figure 2D, lower pie charts) were different for each animal. For example, *Sneathia* was an abundant genus in four animals (mean ranging from 13.7 to 26.2%) but was not present in animal CCA096. *Peptoniphilus* (18.6%) was abundant in CCA096 but present at relative abundances ranging between 4.4 and 8.5% in the other animals. *Porphyromonas* was the most abundant genera in two animals (>20% in CA086 and CCA096), whereas it was only present at an abundance of 3.1% in CB804C. Similarly, *Prevotella*, predominated in the vaginal microbiota in two animals (>20% in BA890I and CB804C) but was only present at abundances of 2 and 9.7% in two others (CBL015 and CA086, respectively). *Peptostreptococcus, Mobiluncus, Anaerococcus*, 1–68, and *Campylobacter* were present in all animals at abundances ranging from 3.6 to 0.5%. Non-identified taxa were also present in the vagina of all animals from an abundance of 1.7–3.6%.

CB804C had the highest number of identified taxa (34) and was also the only one to have detectable *Lactobacillus* in its vaginal microbiota (mean abundance of 2.46%) (Figure 2D, lower pie charts). *Lactobacillus* was present in all 15 collected vaginal samples but varied in abundance from 0.06 to 10.54%. The QIIME analysis was unable to determine the specific species based on the GreenGene database. Further analysis, using RefSeq database (Pruitt et al., 2007) identified the closest *Lactobacillus* species, in terms of sequence homology to be closely related to *Lactobacillus crispatus* (52.13%), *Lactobacillus helveticus* (23.40%), and *Lactobacillus acidophilus* (8.51%) (Table 4).

**Table 4:** Species inference for *Lactobacillus* sequences in CB804C vaginal samples.

***Lactobacillus* species**	**Number of aligned reads**	**% of aligned reads**
*L. crispatus*	49	52.1
*L. helveticus*	22	23.4
*L. acidophilus*	8	8.5
*L. murinus*	4	4.3
*L. reuteri*	4	4.3
*L. delbrueckii*	2	2.1
Unknown	2	2.1
*L. apodemi*	1	1.1
*L. johnsonii*	1	1.1
*L. nagelii*	1	1.1

Overall, we identified a large set of taxa in the cynomolgus macaque rectal and vaginal microbiota. Taxa differed in terms of abundance between the five animals with greater variability in relative abundance and composition in the vaginal microbiota.

### The Vaginal Macaque Microbiota Is More Heterogeneous Than the Rectal Microbiota

**Figure 3:**
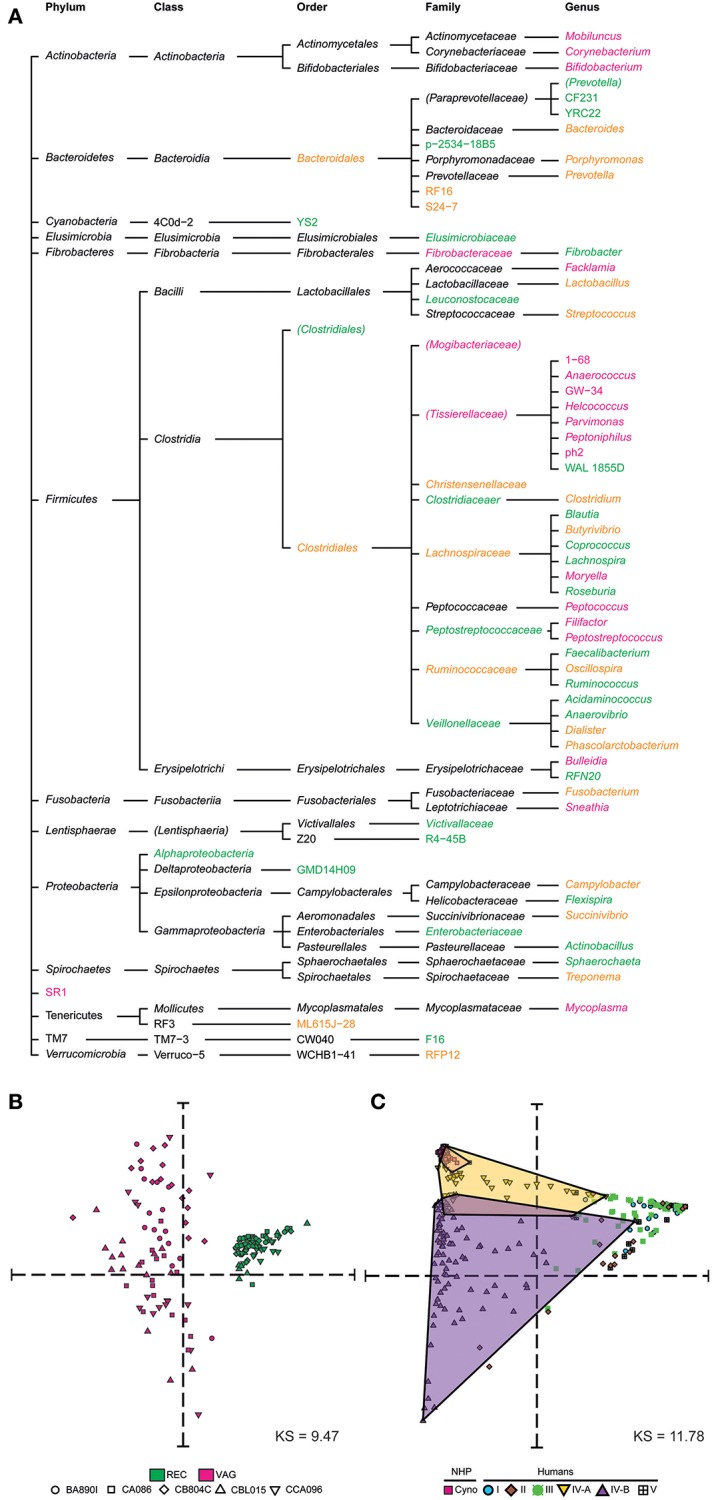
Taxonomic specificities and similarities among rectal and vaginal microbiota. **(A)** Tree representation showing the taxa that were found to be specific to or common between rectal and vaginal samples. Vagina-specific taxa are shown in pink and rectum-specific taxa in green. Taxa found in both the rectum and vagina are shown in orange. **(B)** Multidimensional scaling representation showing the similarities between microbial samples of the dataset in terms of taxa abundance. Each dot in the representation corresponds to a biological sample and the distances between the dots are proportional to the Euclidian distances computed based on their relative taxa abundance. Each symbol represents an animal and each color represents a tissue. **(C)** Multidimensional scaling representation showing the similarities between macaque vaginal microbial samples of the dataset and human vaginal microbial samples from an external dataset (Ravel et al., 2010) in terms of their relative abundance. Five community state types (CST) are defined in humans. CST I, II, III, and V have predominantly *Lactobacillus crispatus, Lactobacillus gasseri, Lactobacillus iners, Lactobacillus jensenii*, respectively. CST IV comprises of a wide array of strict and facultative anaerobes and is further divided into two categories CST IV-A and CST IV-B. Dots are shaped and colored to represent the species. Human samples are also colored and shaped to show the community state types. The Kruskal Stress indicated in each MDS representation corresponds to the percentage of information lost during the dimensionality reduction process.

We next compared the macaque rectal and vaginal microbiota and assessed their similarity and specificity. We created a tree representation (Figure 3A) to compare the taxonomic composition of the macaques' rectal and vaginal microbiota. Taxa are reported and classified with their associated phylum, class, order, family, and genus for clarity. Among the 31 genera identified in rectal samples, 17 (55%) were specific to this tissue. Among the 32 genera identified in vaginal samples, 18 (56%) were specific to this tissue. A total of 14 genera were found both in the rectal and vaginal samples.

We created a MDS representation (Figure 3B) to compare the rectal and vaginal microbial profiles in terms of taxa composition at the genus level. Rectal and vaginal samples were well-segregated. The variability of microbiota composition in the vagina was higher than that of rectum. We qualified sample variabilities by inter-class similarity (IC), which was 0.7334 for the rectum and 2.0351 for the vagina. Overall, vaginal and rectal samples were different in terms of relative taxa abundance, with higher taxonomic composition variability in vaginal samples.

Our analyses revealed that vaginal and rectal microbiota share about half of the detected taxa at the genus level. The cynomolgus macaque vaginal microbiota showed higher variability between animals in both taxa composition and relative abundance.

### Vaginal Cynomolgus Macaque Microbiota Display Similar Composition and Abundance to Woman Vaginal Microbiota Belonging to CST-IV-A

We compared the cynomolgus macaque vaginal microbial profiles with those of humans using a previously published dataset of human vaginal samples collected cross-sectionally (Ravel et al., 2010). This dataset consists of samples from 376 patients assigned to five community state types (CST) designated CST I, II, III, IV-A, IV-B, and CST V. The MDS analysis clearly showed that the cynomolgus macaque vaginal microbiota is most similar, in terms of taxonomic composition and relative abundance, to a subset of women's vaginal microbiota belonging to CST IV-A (Figure 3C).

### The Kinetics of Rectal Microbiota Profiles Are Constant and Similar Between Animals, but the Kinetics of Vaginal Microbiota Profiles Are Highly Variable and Animal-Specific

We next displayed the kinetics of the taxonomic profiles in both rectal and vaginal samples collected within animals over 15 weeks and quantified their variability at each timepoint for each macaque.

**Figure 4:**
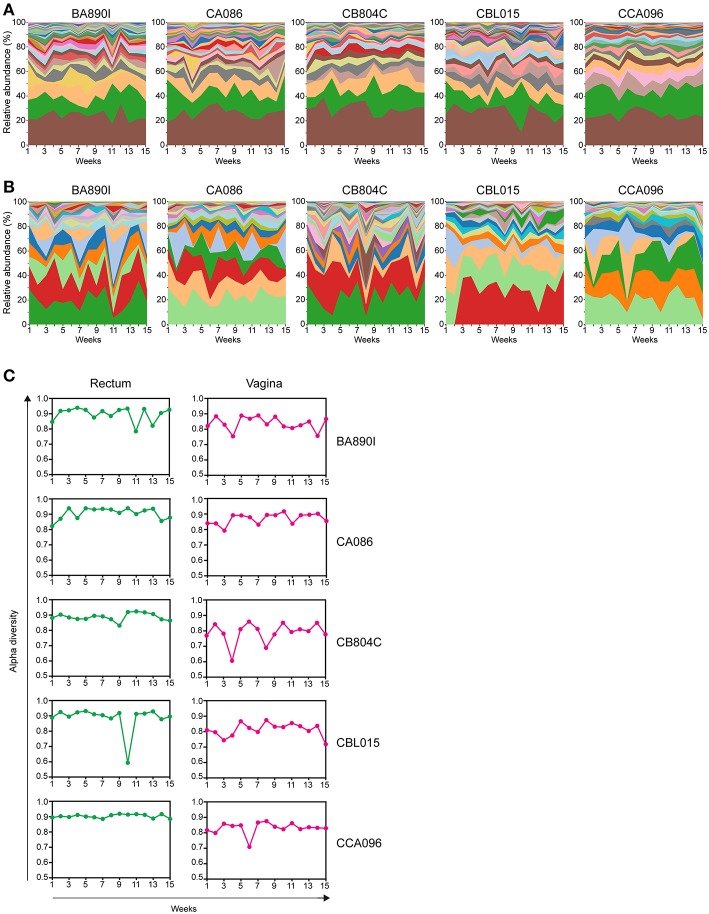
Kinetics of relative taxa abundance. The kinetics of the relative abundance for all identified taxa are shown for each animal, using stream graph representations for rectal **(A)** and vaginal samples **(B)** during the 15 weeks of follow-up. **(C)** The alpha-diversities were computed for each animal and at each time point and are represented for the rectum and vagina. Alpha-diversities were calculated based on the Simpson diversity index (1-dominance).

We generated streamgraph representations for both the rectal (Figure 4A) and vaginal (Figure 4B) microbiota of each macaque to visualize the kinetics of taxonomic profiles. The kinetics were more stable for the rectal microbiota (mean of all 5 animals Jensen-Shannon divergence medians across all time points = 0.0796) than those of the vagina (0.1394). The rank of the most abundant taxa throughout the study was consistent among the different animals in the rectum but was not in the vagina. Several taxa, such as *Lactobacillus*, were not detectable at all timepoints in the vagina.

For the rectum, the number of taxa per timepoint was between 15 and 25, without major differences between timepoints or animals (Supplementary Table 5). For the vagina, the number of taxa per timepoint varied between seven and twenty, with differences between timepoints and animals (Supplementary Table 6).

We quantified the change in alpha diversity, calculated using the Simpson diversity index, for each animal between consecutive timepoints (Figure 4C). The mean alpha diversity was 0.8957 (standard deviation = 0.0465) for the rectal samples whereas the mean alpha diversity was 0.8279 (standard deviation = 0.0529) for the vaginal samples, indicating that the change in diversity is higher in the vagina. The alpha diversity of vaginal samples peaked at various timepoints and was particularly apparent for some animals (e.g., animal CB804C).

These analyses show different kinetics of taxonomic profiles over 15 weeks in both the rectal and vaginal microbiota. In the rectum, similar kinetics were observed between animals, whereas in the vagina, longitudinal profiles were diverse and clearly different between animals. These observations were supported by alpha diversity changes computed between each timepoint for each animal.

### Hormonal Cycles of Female Macaques Influence the Vaginal Microbiota

We then explored whether the lack of stability in vaginal microbiota profiles in cynomolgus macaques is associated with the hormonal cycle. Hormonal cycles were defined by progesterone plasma levels, measured weekly for each animal. This analysis was performed separately for each animal to account for individual variability.

**Figure 5:**
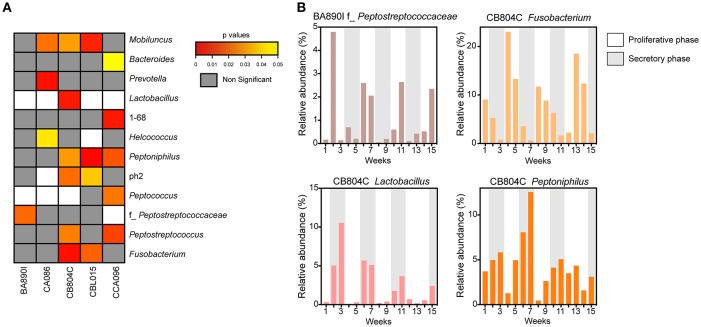
Associations between progesterone levels and taxa abundance in vaginal samples. **(A)** Heatmap representation showing the *p*-values for the association for each taxon, for each animal. The heatmap was restricted to the set of taxa found to be significantly different in abundance between samples taken when progesterone levels were high vs. when they were low. White rectangles represent situations in which differential analyses were not possible because the relative abundance was too low. For each taxon, the name of the genus or family (f_) is indicated. **(B)** Examples of relative taxa abundance and progesterone levels for four significant taxa in animals BA890I and CB804C. The histograms indicate the relative taxa abundance. Progesterone levels are indicated by the gray (≥1 ng/ml) or white (<1 ng/ml) rectangles.

Microbial profiles were classified into two groups, representing low (proliferative phase) or high (secretory phase) progesterone levels. For each animal, we compared the relative abundance of the 47 taxa identified in the vaginal microbiota between the two groups of samples (high vs. low progesterone levels). This differential analysis identified 12 taxa (26% of vaginal taxa) that were differentially abundant in at least one animal according to the progesterone level (Figure 5A), consisting of *Mobiluncus, Bacteroides, Prevotella, Lactobacillus*, 1–68, *Helcococcus, Peptoniphilus*, ph2, *Peptococcus*, f*_Peptostreptococcaceae, Peptostreptococcus*, and *Fusobacterium*.

For example, f_*Peptostreptococcaceae* was associated with hormonal cycling in animal BA890I, as well as *Fusobacterium, Lactobacillus*, and *Peptoniphilus* in animal CB804C (Figure 5B). *Peptostreptococcaceae* was statistically associated (*p* = 0.0209) with the hormonal cycle in animal BA890I, with lower relative abundance in the secretory phase (low progesterone levels). *Fusobacterium* was statistically associated (*p* = 0.0033) with the hormonal cycle in animal CB804C, with a lower abundance in the proliferative phase (high progesterone levels). Conversely *Lactobacillus* (*p* = 0.0058) and *Peptoniphilus* (*p* = 0.0301) in animal CB804C had statistically higher abundance during the secretory phase. Overall, 11 taxa were more abundant during the secretory phase, whereas eight were more abundant in the proliferative phase (Figure 5B and Supplementary Figure 1). In summary, approximately one-quarter of detected vaginal taxa were associated with hormonal cycling.

Thus, time in the menstrual cycle is associated with differential relative abundance of vaginal microbial taxa in macaques. Approximately, a quarter of all identified vaginal taxa had abundances associated with phases of the menstrual cycle in each animal. This proportion dropped to 9% when considering the entire cohort, highlighting considerable individual variability in the bacterial composition and abundance in the cynomolgus macaque vaginal microbiota.

## Discussion

We describe here the composition and dynamics of the rectal and vaginal microbiota in female cynomolgus macaques over 15 weeks capturing at least three menstrual cycles. This is the first time that such a longitudinal study has been reported comparing the microbiota composition of both body sites in the same animals and assessing the hormonal impact.

The major phyla found in the rectum of the cynomolgus macaques are consistent with previous reports on the fecal microbiota of cynomolgus macaques (Seekatz et al., 2013; Cui et al., 2018; Li et al., 2018). The composition of the cynomolgus rectal microbiota is similar to that of high diversity community type II, previously characterized by higher relative abundance of taxa of both *Firmicutes* (*Ruminoccoccae*) and *Bacteroidetes* (*Prevotella*) and lower abundance of *Lactobacillus* (Seekatz et al., 2013). *Firmicutes* and *Bacteroidetes* are also the major phyla found in rectal swabs or feces in humans. The cynomolgus rectal microbiota is somewhat similar to that of enterotype 2 described in humans (Wu et al., 2011), in which *Prevotella* is dominant rather than *Bacteroides*. This enterotype is associated with a low fat, high fiber dietary regimen. X. Li et al. reported that the gut microbiome of cynomolgus macaques is more similar to that of human than those of pig and mouse at the gene level and that the gut microbiota of cynomolgus macaques fed with a high fat, low fiber diet became more similar to the gut microbiota of humans (Li et al., 2018). We did not detect *Actinobacteria*, which is one of the major constituents of the human gastrointestinal tract microbiota (Zoetendal et al., 2008), in the cynomolgus rectal microbiota but rather *Spirochaetes*. Such a lack of rectal *Actinobacteria* does not appear to be related to sample collection or detection issues as *Actinobacteria* were observed in vaginal samples.

The vaginal cynomolgus macaque microbiota is composed of a large variety of anaerobic gram-negative bacteria and unlike humans, is not dominated by *Lactobacillus*. The abundance of *Lactobacillus* (mostly *L. crispatus*) was >1% for only one female macaque in the study. *Lactobacillus* was present in some of the samples in the four other females, but at an abundance of <0.6% (data not filtered, not shown). *L. amylovorus* has been identified at low levels and not in all samples in pigtailed macaques (Spear et al., 2012). In rhesus macaques, previous reports have described vaginal microbiota with few *Lactobacillus* and when present the species was *L. johnsonii* (Yu et al., 2009; Spear et al., 2010). In humans, the most prevalent species are *L. iners, L. crispatus, L. jensenii*, and *L. gasseri* (Spear et al., 2011; Ma et al., 2012).

**Figure 6:**
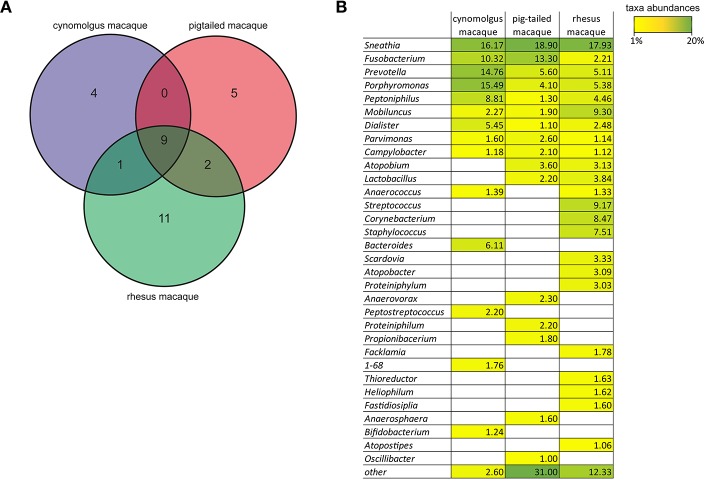
Comparison of the genera found in the vagina of the cynomolgus, pigtailed, and rhesus macaque species. **(A)** Venn diagram showing the specific or common genera present at ≥1% in the vagina of the three different macaque species according to our results for the cynomolgus, (Spear et al., 2010) for the pigtailed, (Spear et al., 2012) for the rhesus. **(B)** Table showing the mean abundance percentages for all genera found in the three macaque species. For the cynomolgus: average number of sequences in the 75 samples from the five animals; For the pigtailed: average number of sequences in 67 samples from 10 animals; For the rhesus: average number of sequences of two sampling time points from 11 animals. Taxa abundance percentages are also indicated by a color-gradient scale ranging from yellow (1%) to green (20%).

It has been previously shown that both Nugent scores and pH values increase as the proportion of non-*Lactobacillus* spp. increases. We did not measure vaginal pH nor determine the Nugent score for the five female cynomolgus macaques included in our study. However, we had the opportunity to measure the vaginal pH and to determine the Nugent scores of female cynomolgus macaques included in parallel studies and housed in the same animal facility. We thus measured the vaginal pH at one timepoint in 12 female cynomolgus macaques, and it ranges between 5.5 and 7.5, with a mean of 6.75. We also measured the vaginal pH of three other females once a week for 1 month. The values were relatively stable over time, ranging from 6.25 to 7.5, and were associated with a Nugent score of 8 for all timepoints. These high pH measures are certainly associated with a lack of copious amount lactic acid produced by *Lactobacillus* spp. when present in high abundance, as lactic acid, with a pKa of 3.86, is a major driver of the low vaginal pH (<4.5) in human. These data combined with our MDS representation clearly show that the vaginal microbial communities in cynomolgus macaques are very close to that of CST IV-A in human: a highly diverse state comprising numerous strict and facultative anaerobes, also associated with elevated Nugent scores and pH (Ravel et al., 2010). This profile has been shown to be strongly associated with increased risk of HIV-1 and other STIs. Most of the vaginal bacterial species of the cynomolgus macaques (such as *Sneathia, Porphyromonas*, and *Prevotella*) are also found in women with symptomatic or asymptomatic BV a condition more common in Black and Hispanic women (Ravel et al., 2010; Kenyon and Osbak, 2015). In contrast, *Gardnerella vaginalis*, another species commonly found in women with BV was not detected in cynomolgus macaques. *G. vaginalis* has been found in pigtailed macaques in a few samples at low levels (Spear et al., 2012). The low level of *Lactobacillus* and the polymicrobial composition of the vaginal microbiota have also been reported in rhesus (Spear et al., 2010) and pigtailed (Spear et al., 2012) macaques. The Venn diagram on Figure 6A shows the main genera that are common or specific to the three macaque species. Nine genera are common to the three species with different abundance percentages but *Sneathia* is the most abundant in all (Figure 6B). Three genera are shared by two species and four are cynomolgus specific, five pigtailed specific and 11 rhesus specific. To date, humans are the only mammals to have vaginal microbiota often dominated by *Lactobacillus* spp., and a very low pH ( ≤ 4.5). The growth of genital *Lactobacillus* spp. is postulated to depend on epithelial cell-produced glycogen (Mirmonsef et al., 2014). It is hypothesized that degradation products of glycogen by human α-amylases provide a selective nutritional advantage to *Lactobacillus* spp., which in turn produce high amount of lactic acid (Boskey et al., 1999; Miller et al., 2016). Elevated estrogen levels promote thickening of the epithelium and the production and accumulation of glycogen in the epithelium (Mirmonsef et al., 2014). Free glycogen levels are significantly negatively associated with both vaginal pH and progesterone in women (Mirmonsef et al., 2016). Humans have higher vaginal concentrations of glycogen than other mammals (Miller et al., 2016) and α-amylase is expressed in the vaginal epithelium (Mirmonsef et al., 2014). The levels of lactic acid and glycogen in the genital fluids of rhesus and pigtailed macaques are lower than those in women (Mirmonsef et al., 2012). Unfortunately, no measures of glycogen, α-amylase activity, and lactic acid levels in the vaginal tract of cynomolgus macaques are available. It has been postulated that high estrogen levels, high *Lactobacillus* spp. relative abundance, and low vaginal pH are difficult to detect in NHP, as they do not cycle continuously (Miller et al., 2016). However, female cynomolgus macaques have an ovarian cycle similar to that of women. Miller et al. reported that mammals other than humans that exhibit continuous cycling have lower vaginal pH during high-estrogen phases, but they never reach the low pH observed in humans. Ravel et al. postulated that vaginal microbiota depleted of *Lactobacillus* spp., could also be considered normal in the absence of sign and symptoms, as beneficial functions may be provided by several types of bacterial composition (Ma et al., 2012). However, this normal state might not be optimal and appear to still carry some risks if exposure to STI agents but evidence supporting this hypothesis are still lacking. On the other hand, it has been shown that genital proinflammatory cytokine levels strongly correlate with diverse community types (Anahtar et al., 2015) and that diverse cervicovaginal bacterial communities are associated with increased HIV-1 acquisition (Gosmann et al., 2017). Furthermore, certain cervicovaginal communities are associated with decreased antiretroviral concentrations in the female genital tract (Donahue Carlson et al., 2017), *G. vaginalis* and other anaerobic bacteria metabolisms significantly deplete tenofovir (Klatt et al., 2017). *G. vaginalis* is poorly or not detectable in the vaginal microbiota of the three macaque species that are the most commonly used to study HIV-1 infection but other anaerobes such as *Prevotella* (Figure 6B) that can also lead to tenofovir depletion are present. This vaginal environment of macaque species should such be consider when analyzing studies on STI acquisition and prevention in these models. Each macaque species has its own advantages and disadvantages. But in term of vaginal microbiota, even if they have their own specificities, they all have a polymicrobial composition with highly diverse communities comprising numerous strict and facultative anaerobes. The differences between the three macaque species are mostly: (i) in term of ovarian cycle as mentioned above; (ii) in term of SIV/SHIV pathogenesis (Ten Haaft et al., 2001; Favre et al., 2009; Antony and Macdonald, 2015); (iii) the susceptibility to infection/coinfection with human pathogens; and (iv) the availability and cost of each model in the different parts of the world.

The inter-individual compositional similarities and stability of the cynomolgus macaque rectal and vaginal microbiota were different. The microbiota composition of the rectum was quite similar between animals, whereas that of the vagina was distinct for each animal. The five animals had the same dietary regimen and were of the same origin, which can explain the low inter-individual variability in the rectum. The bacterial composition of the vagina is influenced by several parameters such, as hormone levels, environmental factors, diet, genetics, sexual behavior, and STIs. The five female macaques included in this study were first or second generation and may have been exposed to different environmental factors.

Diversity was higher in the rectum than in the vagina, and was still associated with higher stability. In the vagina, this lack of stability, with rapid changes, has previously been observed in some women, and the cause of this lack of resilience remains unknown (Gajer et al., 2012). We demonstrated that temporal changes in vaginal composition are statistically associated with progesterone levels, in contrast to those of the rectum microbiota, which were not. However, for each animal, the number and type of vaginal bacteria differentially abundant according to progesterone level were different, which could be explained by the high inter-individual compositional differences observed. Interestingly, some taxa such as *Mobiluncus* or *Peptostreptococcus*, were associated with progesterone levels in some animals but were found in lower abundance in others. Factors not captured in this study and not associated with hormonal cycling may influence the composition and abundance of taxa in the cynomolgus macaque vaginal microbiota. In our study, we observed high relative abundance of *Lactobacillus* spp. in one animal to be associated with high level of progesterone. However, high levels of progesterone were concomitant with lower level of estradiol and followed the major peak of estradiol (Supplementary Figure 2). Thus, it is likely that, similarly to human, in this animal, high levels of progesterone are driving the higher relative abundance of *Lactobacillus* spp.

A better understanding of the relationship between bacterial composition and dynamics, and the influence of the hormonal cycle will help to improve our understanding of the role of vaginal microbiota in STI susceptibility or resistance using animal models such as the cynomolgus macaque. Longitudinal analysis of the vaginal and rectal microbiota in non-human-primate models are necessary for this purpose. Further investigations in macaque models are necessary to understand whether the rectal or vaginal microbiota affect susceptibility to infections and local immune response. Ultimately, access to a well-characterized cynomolgus macaque model in which the vaginal microbiota could be manipulated (local antibiotic treatments and/or local administration of specific bacterial strains) to mimic that of human, will help us to decipher the role of the vaginal microbiota play in the protection against STIs in human.

## Data Availability

The datasets generated for this study can be found in SRA database (Leinonen et al., 2011), PRJNA497378. All raw sequenced paired-end reads, assembled reads, scripts used for the data analysis, and complementary visualization charts of taxa abundances are available on the IDMIT data dissemination platform through the link http://data.idmitcenter.fr/cynobiota/.

## Ethics Statement

Treatment of (NHP) at the CEA complied with French national regulations (CEA authorization A 92-032-02), the Standards for Human Care and Use of Laboratory Animals (OLAW Assurance number #A5826-01), and European Directive 2010/63 (recommendation #9). Experiments were supervised by veterinarians in charge of the animal facility. This study was approved and accredited by the Comité d'Ethique en Expérimentation Animale du CEA (A16-048), and the French Research Ministry.

## Author Contributions

VC, RL, RM, and EM: study conception and design. M-TN, NT, CC, RM, and EM: acquisition of data. M-TN, NT, JR, RM, and EM: analysis and interpretation of data. M-TN, NT, CA, RM, and EM: drafting of manuscript. M-TN, NT, FB, JR, RL, and EM: critical revisions.

### Conflict of Interest Statement

The authors declare that the research was conducted in the absence of any commercial or financial relationships that could be construed as a potential conflict of interest.

## References

[B1] Alfson K. J. Avena L. E. Worwa G. Carrion R. Griffiths A. (2017). Development of a lethal intranasal exposure model of ebola virus in the cynomolgus macaque. Viruses 9:E319. 10.3390/v9110319 29109373 PMC5707526

[B2] Anahtar M. N. Byrne E. H. Doherty K. E. Bowman B. A. Yamamoto H. S. Soumillon M.. (2015). Cervicovaginal bacteria are a major modulator of host inflammatory responses in the female genital tract. Immunity 42, 965–976. 10.1016/j.immuni.2015.04.019 25992865 PMC4461369

[B3] Antonio M. A. Hawes S. E. Hillier S. L. (1999). The identification of vaginal *Lactobacillus* species and the demographic and microbiologic characteristics of women colonized by these species. J. Infect. Dis. 180, 1950–1956. 10.1086/315109 10558952

[B4] Antony J. M. Macdonald K. S. (2015). A critical analysis of the cynomolgus macaque, *Macaca fascicularis*, as a model to test HIV-1/SIV vaccine efficacy. Vaccine 33, 3073–3083. 10.1016/j.vaccine.2014.12.004 25510387

[B5] Becavin C. Tchitchek N. Mintsa-Eya C. Lesne A. Benecke A. (2011). Improving the efficiency of multidimensional scaling in the analysis of high-dimensional data using singular value decomposition. Bioinformatics 27, 1413–1421. 10.1093/bioinformatics/btr143 21421551

[B6] Boratyn G. M. Camacho C. Cooper P. S. Coulouris G. Fong A. Ma N.. (2013). BLAST: a more efficient report with usability improvements. Nucleic Acids Res. 41, W29–33. 10.1093/nar/gkt282 23609542 PMC3692093

[B7] Borgdorff H. Armstrong S. D. Tytgat H. L. Xia D. Ndayisaba G. F. Wastling J. M.. (2016a). Unique insights in the cervicovaginal *Lactobacillus iners* and *L. crispatus* proteomes and their associations with microbiota dysbiosis. PLoS ONE 11:e0150767. 10.1371/journal.pone.0150767 26963809 PMC4786256

[B8] Borgdorff H. Gautam R. Armstrong S. D. Xia D. Ndayisaba G. F. Van Teijlingen N. H.. (2016b). Cervicovaginal microbiome dysbiosis is associated with proteome changes related to alterations of the cervicovaginal mucosal barrier. Mucosal Immunol. 9, 621–633. 10.1038/mi.2015.86 26349657

[B9] Boskey E. R. Telsch K. M. Whaley K. J. Moench T. R. Cone R. A. (1999). Acid production by vaginal flora *in vitro* is consistent with the rate and extent of vaginal acidification. Infect. Immun. 67, 5170–5175. 10496892 10.1128/iai.67.10.5170-5175.1999 PMC96867

[B10] Braundmeier A. G. Lenz K. M. Inman K. S. Chia N. Jeraldo P. Walther-Antonio M. R.. (2015). Individualized medicine and the microbiome in reproductive tract. Front. Physiol. 6:97. 10.3389/fphys.2015.00097 25883569 PMC4381647

[B11] Caporaso J. G. Bittinger K. Bushman F. D. Desantis T. Z. Andersen G. L. Knight R. (2010a). PyNAST: a flexible tool for aligning sequences to a template alignment. Bioinformatics 26, 266–267. 10.1093/bioinformatics/btp636 19914921 PMC2804299

[B12] Caporaso J. G. Kuczynski J. Stombaugh J. Bittinger K. Bushman F. D. Costello E. K.. (2010b). QIIME allows analysis of high-throughput community sequencing data. Nat. Methods 7, 335–336. 10.1038/nmeth.f.303 20383131 PMC3156573

[B13] Clemente J. C. Ursell L. K. Parfrey L. W. Knight R. (2012). The impact of the gut microbiota on human health: an integrative view. Cell 148, 1258–1270. 10.1016/j.cell.2012.01.035 22424233 PMC5050011

[B14] Cribby S. Taylor M. Reid G. (2008). Vaginal microbiota and the use of probiotics. Interdiscip. Perspect. Infect. Dis. 2008:256490. 10.1155/2008/256490 19343185 PMC2662373

[B15] Cui Y. F. Wang F. J. Yu L. Ye H. H. Yang G. B. (2018). Metagenomic comparison of the rectal microbiota between rhesus macaques (*Macaca mulatta*) and cynomolgus macaques (*Macaca fascicularis*). Zool. Res. 40, 89–93. 10.24272/j.issn.2095-8137.2018.061. 30127329 PMC6378564

[B16] Desantis T. Z. Hugenholtz P. Larsen N. Rojas M. Brodie E. L. Keller K.. (2006). Greengenes, a chimera-checked 16S rRNA gene database and workbench compatible with ARB. Appl. Environ. Microbiol. 72, 5069–5072. 10.1128/AEM.03006-05 16820507 PMC1489311

[B17] Donahue Carlson R. Sheth A. N. Read T. D. Frisch M. B. Mehta C. C. Martin A.. (2017). The female genital tract microbiome is associated with vaginal antiretroviral drug concentrations in human immunodeficiency virus-infected women on antiretroviral therapy. J. Infect. Dis. 216, 990–999. 10.1093/infdis/jix420 29029138 PMC5853913

[B18] Edgar R. C. (2010). Search and clustering orders of magnitude faster than BLAST. Bioinformatics 26, 2460–2461. 10.1093/bioinformatics/btq461 20709691

[B19] Eschenbach D. A. Davick P. R. Williams B. L. Klebanoff S. J. Young-Smith K. Critchlow C. M.. (1989). Prevalence of hydrogen peroxide-producing *Lactobacillus* species in normal women and women with bacterial vaginosis. J. Clin. Microbiol. 27, 251–256. 2915019 10.1128/jcm.27.2.251-256.1989 PMC267286

[B20] Favre D. Lederer S. Kanwar B. Ma Z. M. Proll S. Kasakow Z.. (2009). Critical loss of the balance between Th17 and T regulatory cell populations in pathogenic SIV infection. PLoS Pathog. 5:e1000295. 10.1371/journal.ppat.1000295 19214220 PMC2635016

[B21] Ferreira V. H. Kafka J. K. Kaushic C. (2014). Influence of common mucosal co-factors on HIV infection in the female genital tract. Am. J. Reprod. Immunol. 71, 543–554. 10.1111/aji.12221 24617528

[B22] Fredricks D. N. Fiedler T. L. Marrazzo J. M. (2005). Molecular identification of bacteria associated with bacterial vaginosis. N. Engl. J. Med. 335, 1899–1911. 10.1056/NEJMoa043802 16267321

[B23] Gajer P. Brotman R. M. Bai G. Sakamoto J. Schutte U. M. Zhong X.. (2012). Temporal dynamics of the human vaginal microbiota. Sci. Transl. Med. 4, 132–152. 10.1126/scitranslmed.3003605 22553250 PMC3722878

[B24] Ganesh B. P. Versalovic J. (2015). Luminal conversion and immunoregulation by probiotics. Front. Pharmacol. 6:269. 10.3389/fphar.2015.00269 26617521 PMC4641912

[B25] Giorgi A. Torriani S. Dellaglio F. Stola E. Bernuzzi L. (1987). Identification of vaginal lactobacilli from asymptomatic women. Microbiologica 10, 377–384. 3695985

[B26] Gosmann C. Anahtar M. N. Handley S. A. Farcasanu M. Abu-Ali G. Bowman B. A.. (2017). *Lactobacillus*-deficient cervicovaginal bacterial communities are associated with increased HIV acquisition in young South African women. Immunity 46, 29–37. 10.1016/j.immuni.2016.12.013 28087240 PMC5270628

[B27] Homayouni A. Ziyadi S. Mohammad-Alizadeh-Charandabi S. Ghalibaf M. Mortazavian A. M. Mehrabany E. V. (2014). Effects of probiotics on the recurrence of bacterial vaginosis: a review. J. Low. Genit. Tract Dis. 18, 79–86. 10.1097/LGT.0b013e31829156ec 24299970

[B28] Kenyon C. Osbak K. (2015). Sexual networks, HIV, race and bacterial vaginosis. AIDS 29, 641–642. 10.1097/QAD.0000000000000566 25710290

[B29] Klatt N. R. Cheu R. Birse K. Zevin A. S. Perner M. Noël-Romas L.. (2017). Vaginal bacteria modify HIV tenofovir microbicide efficacy in African women. Science 356, 938–945. 10.1126/science.aai9383 28572388

[B30] Klindworth A. Pruesse E. Schweer T. Peplies J. Quast C. Horn M.. (2013). Evaluation of general 16S ribosomal RNA gene PCR primers for classical and next-generation sequencing-based diversity studies. Nucleic Acids Res. 41:e1. 10.1093/nar/gks808 22933715 PMC3592464

[B31] Landman C. Quevrain E. (2016). Gut microbiota: description, role and pathophysiologic implications. Rev. Med. Interne 37, 418–423. 10.1016/j.revmed.2015.12.012 26749318

[B32] Leinonen R. Sugawara H. Shumway M. International Nucleotide Sequence Database C. (2011). The sequence read archive. Nucleic Acids Res. 39, D19–21. 10.1093/nar/gkq1019 21062823 PMC3013647

[B33] Li X. Liang S. Xia Z. Qu J. Liu H. Liu C.. (2018). Establishment of a *Macaca fascicularis* gut microbiome gene catalog and comparison with the human, pig, and mouse gut microbiomes. Gigascience 7, 1–10. 10.1093/gigascience/giy100 30137359 PMC6137240

[B34] Ma B. Forney L. J. Ravel J. (2012). Vaginal microbiome: rethinking health and disease. Annu. Rev. Microbiol. 66, 371–389. 10.1146/annurev-micro-092611-150157 22746335 PMC3780402

[B35] Magoc T. Salzberg S. L. (2011). FLASH: fast length adjustment of short reads to improve genome assemblies. Bioinformatics 27, 2957–2963. 10.1093/bioinformatics/btr507 21903629 PMC3198573

[B36] Manuzak J. A. Hensley-Mcbain T. Zevin A. S. Miller C. Cubas R. Agricola B.. (2016). Enhancement of microbiota in healthy macaques results in beneficial modulation of mucosal and systemic immune function. J. Immunol. 196, 2401–2409. 10.4049/jimmunol.1502470 26826246 PMC4761491

[B37] Martin M. (2011). Cutadapt removes adapter sequences from high-throughput sequencing reads. EMBnet.journal 17, 10–12. 10.14806/ej.17.1.200

[B38] Miller E. A. Beasley D. E. Dunn R. R. Archie E. A. (2016). Lactobacilli dominance and vaginal pH: why is the human vaginal microbiome unique? Front. Microbiol. 7:1936. 10.3389/fmicb.2016.01936 28008325 PMC5143676

[B39] Mirmonsef P. Gilbert D. Veazey R. S. Wang J. Kendrick S. R. Spear G. T. (2012). A comparison of lower genital tract glycogen and lactic acid levels in women and macaques: implications for HIV and SIV susceptibility. AIDS Res. Hum. Retroviruses 28, 76–81. 10.1089/aid.2011.0071 21595610 PMC3251838

[B40] Mirmonsef P. Hotton A. L. Gilbert D. Burgad D. Landay A. Weber K. M.. (2014). Free glycogen in vaginal fluids is associated with *Lactobacillus* colonization and low vaginal pH. PLoS ONE 9:e102467. 10.1371/journal.pone.0102467 25033265 PMC4102502

[B41] Mirmonsef P. Hotton A. L. Gilbert D. Gioia C. J. Maric D. Hope T. J.. (2016). Glycogen levels in undiluted genital fluid and their relationship to vaginal pH, estrogen, and progesterone. PLoS ONE 11:e0153553. 10.1371/journal.pone.0153553 27093050 PMC4836725

[B42] Paulson J. N. Stine O. C. Bravo H. C. Pop M. (2013). Differential abundance analysis for microbial marker-gene surveys. Nat. Methods 10, 1200–1202. 10.1038/nmeth.2658 24076764 PMC4010126

[B43] Petrova M. I. Mathys L. Lebeer S. Noppen S. Van Damme E. J. Tanaka H.. (2013). Inhibition of infection and transmission of HIV-1 and lack of significant impact on the vaginal commensal lactobacilli by carbohydrate-binding agents. J. Antimicrob. Chemother. 68, 2026–2037. 10.1093/jac/dkt152 23640125

[B44] Pruitt K. D. Tatusova T. Maglott D. R. (2007). NCBI reference sequences (RefSeq): a curated non-redundant sequence database of genomes, transcripts and proteins. Nucleic Acids Res. 35, D61–65. 10.1093/nar/gkl842 17130148 PMC1716718

[B45] Ravel J. Gajer P. Abdo Z. Schneider G. M. Koenig S. S. K. Mcculle S. L.. (2010). Vaginal microbiome of reproductive-age women. PNAS 108, 4680–4687. 10.1073/pnas.1002611107 20534435 PMC3063603

[B46] Seekatz A. M. Panda A. Rasko D. A. Toapanta F. R. Eloe-Fadrosh E. A. Khan A. Q.. (2013). Differential response of the cynomolgus macaque gut microbiota to *Shigella* infection. PLoS ONE 8:e64212. 10.1371/journal.pone.0064212 23755118 PMC3673915

[B47] Sharpe S. A. White A. D. Sibley L. Gleeson F. Hall G. A. Basaraba R. J.. (2017). An aerosol challenge model of tuberculosis in Mauritian cynomolgus macaques. PLoS ONE 12:e0171906. 10.1371/journal.pone.0171906 28273087 PMC5342172

[B48] Spear G. T. Gilbert D. Landay A. L. Zariffard R. French A. L. Patel P.. (2011). Pyrosequencing of the genital microbiotas of HIV-seropositive and -seronegative women reveals *Lactobacillus iners* as the predominant *Lactobacillus* species. Appl. Environ. Microbiol. 77, 378–381. 10.1128/AEM.00973-10 21075899 PMC3019699

[B49] Spear G. T. Gilbert D. Sikaroodi M. Doyle L. Green L. Gillevet P. M.. (2010). Identification of rhesus macaque genital microbiota by 16S pyrosequencing shows similarities to human bacterial vaginosis: implications for use as an animal model for HIV vaginal infection. AIDS Res. Hum. Retroviruses 26, 193–200. 10.1089/aid.2009.0166 20156101 PMC2835387

[B50] Spear G. T. Kersh E. Guenthner P. Vishwanathan S. A. Gilbert D. Zariffard M. R.. (2012). Longitudinal assessment of pigtailed macaque lower genital tract microbiota by pyrosequencing reveals dissimilarity to the genital microbiota of healthy humans. AIDS Res. Hum. Retroviruses 28, 1244–1249. 10.1089/aid.2011.0382 22264029 PMC3448102

[B51] Tachedjian G. O'hanlon D. E. Ravel J. (2018). The implausible “*in vivo*” role of hydrogen peroxide as an antimicrobial factor produced by vaginal microbiota. Microbiome 6:29. 10.1186/s40168-018-0418-3 29409534 PMC5801833

[B52] Ten Haaft P. Almond N. Biberfeld G. Cafaro A. Cranage M. Ensoli B.. (2001). Comparison of early plasma RNA loads in different macaque species and the impact of different routes of exposure on SIV/SHIV infection. J. Med. Primatol. 30, 207–214. 10.1034/j.1600-0684.2001.d01-54.x 11555139

[B53] Vujkovic-Cvijin I. Dunham R. M. Iwai S. Maher M. C. Albright R. G. Broadhurst M. J.. (2013). Dysbiosis of the gut microbiota is associated with HIV disease progression and tryptophan catabolism. Sci. Transl. Med. 5:193ra191. 10.1126/scitranslmed.3006438 23843452 PMC4094294

[B54] Wang Q. Garrity G. M. Tiedje J. M. Cole J. R. (2007). Naive Bayesian classifier for rapid assignment of rRNA sequences into the new bacterial taxonomy. Appl. Environ. Microbiol. 73, 5261–5267. 10.1128/AEM.00062-07 17586664 PMC1950982

[B55] Weinbauer G. F. Niehoff M. Niehaus M. Srivastav S. Fuchs A. Van Esch E.. (2008). Physiology and endocrinology of the ovarian cycle in macaques. Toxicol. Pathol. 36, 7S−23S. 10.1177/0192623308327412 20852722 PMC2939751

[B56] Wu G. D. Chen J. Hoffmann C. Bittinger K. Chen Y.-Y. Keilbaugh S. A.. (2011). Linking long-term dietary patterns with gut microbial enterotypes. Science 334, 105–108. 10.1126/science.1208344 21885731 PMC3368382

[B57] Yu R. R. Cheng A. T. Lagenaur L. A. Huang W. Weiss D. E. Treece J.. (2009). A Chinese rhesus macaque (*Macaca mulatta*) model for vaginal *Lactobacillus* colonization and live microbicide development. J. Med. Primatol. 38, 125–136. 10.1111/j.1600-0684.2008.00316.x 19367737 PMC4422182

[B58] Ziklo N. Huston W. M. Taing K. Katouli M. Timms P. (2016). *In vitro* rescue of genital strains of *Chlamydia trachomatis* from interferon-gamma and tryptophan depletion with indole-positive, but not indole-negative *Prevotella* spp. BMC Microbiol. 16:286 10.1186/s12866-016-0903-4 27914477 PMC5135834

[B59] Zoetendal E. G. Rajilic-Stojanovic M. De Vos W. M. (2008). High-throughput diversity and functionality analysis of the gastrointestinal tract microbiota. Gut 57, 1605–1615. 10.1136/gut.2007.133603 18941009

