# Supplementary material for: Dynamics of Vaginal and Rectal Microbiota Over Several Menstrual Cycles in Female Cynomolgus Macaques
Source: Front Cell Infect Microbiol. 2019 Jun 12;9:188. doi: 10.3389/fcimb.2019.00188 (PMC6582644; doi:10.3389/fcimb.2019.00188)
Supplement: Supplementary file 1 [file Data_Sheet_1.PDF]

Dynamics of Vaginal and Rectal Microbiota over Several Menstrual Cycles in Female Cynomolgus Macaques

Supplemental Materials

Marie-Thérèse Nugeyre[1,2,*], Nicolas Tchitchek[1,*], Cindy Adapen[1], Claude Cannou[1,2], Vanessa Contreras[1], Fahd Benjelloun[1,2], Jacques Ravel[3], Roger Le Grand[1], Romain Marlin[1], and Elisabeth Menu[1,2,¶]

[1] CEA, Université Paris-Sud, Inserm, U1184 « Immunology of viral infections and autoimmune diseases » (IMVA), IDMIT Department, IBFJ, Fontenay-Aux-Roses, France

[2] MISTIC group, Department of Virology, Institut Pasteur, Paris, France

[3] Institute for Genome Sciences and Department of Microbiology and Immunology, University of Maryland School of Medicine, Baltimore, USA.

* These two authors equally contributed to the work.

¶ Correspondence to Dr. Elisabeth Menu, E-mail: elisabeth.menu@cea.fr

Supplementary Table 1 – Relative abundances of microbial taxa in macaque rectal samples at the phylum level.

	Animals					
Phylum	ALL	BA890I	CA086	CB804C	CBL015	CCA096
Firmicutes	54.73	54.63	54.85	58.82	51.75	53.58
Bacteroidetes	31.34	28.93	31.40	26.72	34.30	35.34
Fusobacteria	0.07		0.17		0.21	
Proteobacteria	5.92	8.78	5.38	3.03	7.91	4.50
Other	2.62	2.65	2.26	2.80	2.97	2.42
Actinobacteria						
Spirochaetes	3.57	3.30	3.48	5.35	2.23	3.50
Verrucomicrobia	0.72	0.99	0.98	1.01	0.63	
Tenericutes	0.22		0.64	0.47		
SR1						
Lentisphaerae	0.27		0.38	0.34		0.65
TM7	0.20			0.98		
Cyanobacteria	0.14	0.72				
Fibrobacteres	0.10			0.49		
Elusimicrobia	0.09		0.46			

Supplementary Table 2 – Relative abundances of microbial taxa in macaque vaginal samples at the phylum level.

	VAG					
Phylum	ALL	BA890I	CA086	CB804C	CBL015	CCA096
Firmicutes	27.65	27.45	22.59	27.80	21.76	38.65
Bacteroidetes	36.68	38.70	41.25	29.93	28.19	45.32
Fusobacteria	26.50	24.94	28.80	29.32	40.24	9.19
Proteobacteria	1.25	0.84	1.77	0.62	2.06	0.96
Other	2.60	3.55	2.97	2.66	1.69	2.15
Actinobacteria	4.35	3.83	1.74	8.32	4.13	3.73
Spirochaetes	0.19		0.35	0.59		
Verrucomicrobia	0.04			0.19		
Tenericutes	0.36	0.69	0.53	0.57		
SR1	0.39				1.93	

Supplementary Table 3 – Relative abundances of microbial taxa in macaque rectal samples.

phylum	class	order	family	genus	BA890I	CA086	CB804C	CBL015	CCA096	all samples
Bacteroidetes	Bacteroidia	Bacteroidales			5.58	5.93	5.16	6.51	3.07	5.25
Bacteroidetes	Bacteroidia	Bacteroidales	[Paraprevotellaceae]	[Prevotella]	0.44	0.76	0.00	0.80	1.16	0.63
Bacteroidetes	Bacteroidia	Bacteroidales	[Paraprevotellaceae]	CF231	0.00	1.06	0.00	0.74	0.00	0.36
Bacteroidetes	Bacteroidia	Bacteroidales	[Paraprevotellaceae]	YRC22	0.00	0.71	0.00	0.00	0.00	0.14
Bacteroidetes	Bacteroidia	Bacteroidales	Bacteroidaceae	Bacteroides	0.00	0.00	0.00	2.88	0.38	0.65
Bacteroidetes	Bacteroidia	Bacteroidales	p-2534-18B5		1.35	0.00	0.32	2.13	0.27	0.81
Bacteroidetes	Bacteroidia	Bacteroidales	Porphyromonadaceae	Porphyromonas	0.43	0.27	0.00	0.00	0.22	0.18
Bacteroidetes	Bacteroidia	Bacteroidales	Prevotellaceae	Prevotella	15.58	17.45	14.83	14.14	20.62	16.52
Bacteroidetes	Bacteroidia	Bacteroidales	RF16		2.24	1.75	1.09	1.47	0.35	1.38
Bacteroidetes	Bacteroidia	Bacteroidales	S24-7		3.30	3.47	5.32	5.62	9.28	5.40
Cyanobacteria	4C0d-2	YS2			0.72	0.00	0.00	0.00	0.00	0.14
Elusimicrobia	Elusimicrobia	Elusimicrobiales	Elusimicrobiaceae		0.00	0.46	0.00	0.00	0.00	0.09
Fibrobacteres	Fibrobacteria	Fibrobacterales	Fibrobacteraceae	Fibrobacter	0.00	0.00	0.49	0.00	0.00	0.10
Firmicutes	Bacilli	Lactobacillales	Lactobacillaceae	Lactobacillus	0.00	2.10	0.00	0.92	0.30	0.66
Firmicutes	Bacilli	Lactobacillales	Leuconostocaceae		0.31	0.00	0.00	0.00	0.00	0.06
Firmicutes	Bacilli	Lactobacillales	Streptococcaceae	Streptococcus	0.30	1.93	0.00	1.64	0.44	0.86
Firmicutes	Clostridia	Clostridiales			11.10	9.92	11.11	8.19	5.75	9.21
Firmicutes	Clostridia	Clostridiales	[Tissierellaceae]	WAL_1855D	0.18	0.00	0.00	0.00	0.00	0.04
Firmicutes	Clostridia	Clostridiales	Christensenellaceae		2.03	2.02	3.59	1.07	0.67	1.88
Firmicutes	Clostridia	Clostridiales	Clostridiaceae		1.59	0.98	0.63	0.44	0.92	0.91
Firmicutes	Clostridia	Clostridiales	Clostridiaceae	Clostridium	0.56	0.64	0.37	0.61	0.00	0.44
Firmicutes	Clostridia	Clostridiales	Lachnospiraceae		2.27	2.67	2.06	2.36	6.17	3.11
Firmicutes	Clostridia	Clostridiales	Lachnospiraceae	Blautia	0.56	0.00	0.56	0.50	0.00	0.33
Firmicutes	Clostridia	Clostridiales	Lachnospiraceae	Butyrivibrio	0.00	0.00	0.64	0.00	0.00	0.13
Firmicutes	Clostridia	Clostridiales	Lachnospiraceae	Coprococcus	1.14	1.13	0.62	1.01	2.59	1.30
Firmicutes	Clostridia	Clostridiales	Lachnospiraceae	Lachnospira	0.31	0.00	0.34	0.41	1.00	0.41
Firmicutes	Clostridia	Clostridiales	Lachnospiraceae	Roseburia	0.98	0.00	0.00	1.13	2.53	0.93
Firmicutes	Clostridia	Clostridiales	Ruminococcaceae		24.13	26.20	30.02	26.81	24.77	26.39
Firmicutes	Clostridia	Clostridiales	Ruminococcaceae	Faecalibacterium	1.09	1.07	1.09	0.00	0.00	0.65
Firmicutes	Clostridia	Clostridiales	Ruminococcaceae	Oscillospira	2.71	2.94	3.83	3.68	4.34	3.50
Firmicutes	Clostridia	Clostridiales	Ruminococcaceae	Ruminococcus	3.21	2.38	1.93	1.15	2.28	2.19
Firmicutes	Clostridia	Clostridiales	Veillonellaceae		0.17	0.00	0.00	0.00	0.39	0.11
Firmicutes	Clostridia	Clostridiales	Veillonellaceae	Acidaminococcus	0.00	0.00	0.00	0.20	0.00	0.04
Firmicutes	Clostridia	Clostridiales	Veillonellaceae	Anaerovibrio	0.49	0.00	0.00	0.47	0.48	0.29
Firmicutes	Clostridia	Clostridiales	Veillonellaceae	Dialister	0.99	0.49	1.14	0.45	0.96	0.81
Firmicutes	Clostridia	Clostridiales	Veillonellaceae	Phascolarctobacterium	0.00	0.00	0.88	0.70	0.00	0.32
Firmicutes	Clostridia	[Clostridiales]			0.00	0.37	0.00	0.00	0.00	0.07
Firmicutes	Erysipelotrichi	Erysipelotrichales	Erysipelotrichaceae	RFN20	0.52	0.00	0.00	0.00	0.00	0.10
Fusobacteria	Fusobacteriia	Fusobacteriales	Fusobacteriaceae	Fusobacterium	0.00	0.17	0.00	0.21	0.00	0.07
Lentisphaerae	[Lentisphaeria]	Victivallales	Victivallaceae		0.00	0.38	0.00	0.00	0.65	0.21
Lentisphaerae	[Lentisphaeria]	Z20	R4-45B		0.00	0.00	0.34	0.00	0.00	0.07
Proteobacteria	Alphaproteobacteria				1.02	0.00	0.00	0.00	0.00	0.20
Proteobacteria	Deltaproteobacteria	GMD14H09			0.00	2.01	0.90	2.81	1.42	1.43
Proteobacteria	Epsilonproteobacteria	Campylobacterales	Campylobacteraceae	Campylobacter	0.20	0.00	0.74	0.00	0.00	0.19
Proteobacteria	Epsilonproteobacteria	Campylobacterales	Helicobacteraceae	Flexispira	6.17	2.40	1.39	0.31	0.00	2.05
Proteobacteria	Gammaproteobacteria	Aeromonadales	Succinivibrionaceae	Succinivibrio	1.38	0.97	0.00	4.41	2.83	1.92
Proteobacteria	Gammaproteobacteria	Enterobacteriales	Enterobacteriaceae		0.00	0.00	0.00	0.38	0.00	0.08
Proteobacteria	Gammaproteobacteria	Pasteurellales	Pasteurellaceae	Actinobacillus	0.00	0.00	0.00	0.00	0.26	0.05
Spirochaetes	Spirochaetes	Sphaerochaetales	Sphaerochaetaceae	Sphaerochaeta	0.00	0.00	1.02	0.00	0.00	0.20
Spirochaetes	Spirochaetes	Spirochaetales	Spirochaetaceae	Treponema	3.30	3.48	4.33	2.23	3.50	3.37
Tenericutes	RF3	ML615J-28			0.00	0.64	0.47	0.00	0.00	0.22
TM7	TM7-3	CW040	F16		0.00	0.00	0.98	0.00	0.00	0.20
Verrucomicrobia	Verruco-5	WCHB1-41	RFP12		0.99	0.98	1.01	0.63	0.00	0.72
Other	Other	Other	Other	Other	2.65	2.26	2.80	2.97	2.42	2.62

Supplementary Table 4 – Relative abundances of microbial taxa in macaque vaginal samples.

phylum	class	order	family	genus	BA890I	CA086	CB804C	CBL015	CCA096	all samples
Actinobacteria	Actinobacteria	Actinomycetales	Actinomycetaceae	Mobiluncus	1.66	1.74	1.23	3.64	3.08	2.27
Actinobacteria	Actinobacteria	Actinomycetales	Corynebacteriaceae	Corynebacterium	0.38	0.00	0.00	0.00	0.00	0.08
Actinobacteria	Actinobacteria	Bifidobacteriales	Bifidobacteriaceae		0.00	0.00	3.84	0.00	0.00	0.77
Actinobacteria	Actinobacteria	Bifidobacteriales	Bifidobacteriaceae	Bifidobacterium	1.79	0.00	3.25	0.49	0.65	1.24
Bacteroidetes	Bacteroidia	Bacteroidales			0.00	0.00	0.69	0.00	0.00	0.14
Bacteroidetes	Bacteroidia	Bacteroidales	Bacteroidaceae	Bacteroides	6.89	9.26	0.91	6.52	6.94	6.11
Bacteroidetes	Bacteroidia	Bacteroidales	Porphyromonadaceae	Porphyromonas	11.71	22.34	3.11	19.64	20.66	15.49
Bacteroidetes	Bacteroidia	Bacteroidales	Prevotellaceae	Prevotella	20.10	9.65	24.29	2.02	17.72	14.76
Bacteroidetes	Bacteroidia	Bacteroidales	RF16		0.00	0.00	0.48	0.00	0.00	0.10
Bacteroidetes	Bacteroidia	Bacteroidales	S24-7		0.00	0.00	0.46	0.00	0.00	0.09
Firmicutes	Bacilli	Lactobacillales	Aerococcaceae	Facklamia	0.28	0.00	0.00	0.00	0.00	0.06
Firmicutes	Bacilli	Lactobacillales	Lactobacillaceae	Lactobacillus	0.00	0.00	2.46	0.00	0.00	0.49
Firmicutes	Bacilli	Lactobacillales	Streptococcaceae	Streptococcus	0.00	0.00	0.54	0.19	0.64	0.27
Firmicutes	Clostridia	Clostridiales			0.00	0.00	1.65	0.00	0.21	0.37
Firmicutes	Clostridia	Clostridiales	[Mogibacteriaceae]		0.00	0.39	0.00	0.00	0.00	0.08
Firmicutes	Clostridia	Clostridiales	[Tissierellaceae]		0.00	0.00	0.00	0.00	0.96	0.19
Firmicutes	Clostridia	Clostridiales	[Tissierellaceae]	1-68	0.94	3.14	0.61	1.13	2.97	1.76
Firmicutes	Clostridia	Clostridiales	[Tissierellaceae]	Anaerococcus	1.40	0.49	1.30	0.67	3.08	1.39
Firmicutes	Clostridia	Clostridiales	[Tissierellaceae]	GW-34	0.00	0.00	0.00	0.18	0.00	0.04
Firmicutes	Clostridia	Clostridiales	[Tissierellaceae]	Helcococcus	0.13	0.29	0.19	0.00	0.32	0.19
Firmicutes	Clostridia	Clostridiales	[Tissierellaceae]	Parvimonas	1.82	2.51	0.87	2.81	0.00	1.60
Firmicutes	Clostridia	Clostridiales	[Tissierellaceae]	Peptoniphilus	8.50	7.58	4.41	5.02	18.56	8.81
Firmicutes	Clostridia	Clostridiales	[Tissierellaceae]	ph2	1.80	0.00	0.82	0.59	0.75	0.79
Firmicutes	Clostridia	Clostridiales	Christensenellaceae		0.00	0.00	0.33	0.00	0.00	0.07
Firmicutes	Clostridia	Clostridiales	Clostridiaceae	Clostridium	0.00	0.00	0.00	0.00	0.72	0.14
Firmicutes	Clostridia	Clostridiales	Lachnospiraceae		0.00	0.00	0.27	0.30	0.00	0.11
Firmicutes	Clostridia	Clostridiales	Lachnospiraceae	Butyrivibrio	0.00	0.30	0.00	0.00	0.00	0.06
Firmicutes	Clostridia	Clostridiales	Lachnospiraceae	Moryella	1.15	1.03	0.00	0.00	0.00	0.44
Firmicutes	Clostridia	Clostridiales	Peptococcaceae	Peptococcus	0.00	0.00	0.00	0.39	0.83	0.24
Firmicutes	Clostridia	Clostridiales	Peptostreptococcaceae		1.17	1.33	0.88	2.26	0.00	1.13
Firmicutes	Clostridia	Clostridiales	Peptostreptococcaceae	Filifactor	0.54	0.00	0.81	0.00	0.75	0.42
Firmicutes	Clostridia	Clostridiales	Peptostreptococcaceae	Peptostreptococcus	1.94	1.61	2.45	3.73	1.29	2.20
Firmicutes	Clostridia	Clostridiales	Ruminococcaceae		0.00	0.00	3.59	0.65	0.17	0.88
Firmicutes	Clostridia	Clostridiales	Ruminococcaceae	Oscillospira	0.00	0.00	0.43	0.00	0.00	0.09
Firmicutes	Clostridia	Clostridiales	Veillonellaceae	Dialister	7.34	3.92	6.04	3.33	6.60	5.45
Firmicutes	Clostridia	Clostridiales	Veillonellaceae	Phascolarctobacterium	0.00	0.00	0.15	0.00	0.00	0.03
Firmicutes	Erysipelotrichi	Erysipelotrichales	Erysipelotrichaceae	Bulleidia	0.45	0.00	0.00	0.50	0.82	0.35
Fusobacteria	Fusobacteria	Fusobacteriales	Fusobacteriaceae	Fusobacterium	5.36	15.11	7.95	14.00	9.19	10.32
Fusobacteria	Fusobacteria	Fusobacteriales	Leptotrichiaceae	Sneathia	19.57	13.69	21.37	26.24	0.00	16.17
Proteobacteria	Epsilonproteobacteria	Campylobacterales	Campylobacteraceae	Campylobacter	0.84	1.77	0.62	1.82	0.86	1.18
Proteobacteria	ammaproteobacteria	Aeromonadales	Succinivibrionaceae	Succinivibrio	0.00	0.00	0.00	0.24	0.10	0.07
Spirochaetes	Spirochaetes	Spirochaetales	Spirochaetaceae	Treponema	0.00	0.35	0.59	0.00	0.00	0.19
SR1					0.00	0.00	0.00	1.93	0.00	0.39
Tenericutes	Mollicutes	Mycoplasmatales	Mycoplasmataceae	Mycoplasma	0.69	0.53	0.40	0.00	0.00	0.32
Tenericutes	RF3	ML615J-28			0.00	0.00	0.17	0.00	0.00	0.03
Verrucomicrobia	Verruco-5	WCHB1-41	RFP12		0.00	0.00	0.19	0.00	0.00	0.04
Other	Other	Other	Other	Other	3.55	2.97	2.66	1.69	2.15	2.60

Supplementary Table 5 – Numbers of identified taxa in macaque rectal samples (≥ 1% of sequences)

Week	BA890I	CA086	CB804C	CBL015	CCA096
1	17	19	17	18	20
2	18	17	16	15	15
3	25	16	15	18	17
4	18	18	15	14	15
5	19	17	16	17	18
6	17	18	13	19	15
7	17	16	13	18	17
8	16	18	15	15	14
9	19	18	16	18	15
10	15	17	18	15	15
11	20	18	15	17	19
12	15	21	15	17	16
13	20	20	12	18	16
14	18	14	14	17	19
15	20	14	15	14	15

Supplementary Table 6 – Numbers of identified taxa in macaque vaginal samples (≥ 1% of sequences)

Week	BA890I	CA086	CB804C	CBL015	CCA096
1	11	16	11	11	10
2	17	15	14	13	12
3	11	10	19	14	12
4	15	13	10	13	9
5	13	11	12	13	10
6	13	14	16	14	7
7	15	11	11	14	12
8	14	13	16	15	14
9	10	11	17	12	10
10	10	15	20	15	12
11	16	13	16	13	13
12	13	12	13	13	13
13	11	12	10	12	10
14	12	15	19	16	10
15	14	14	15	12	14

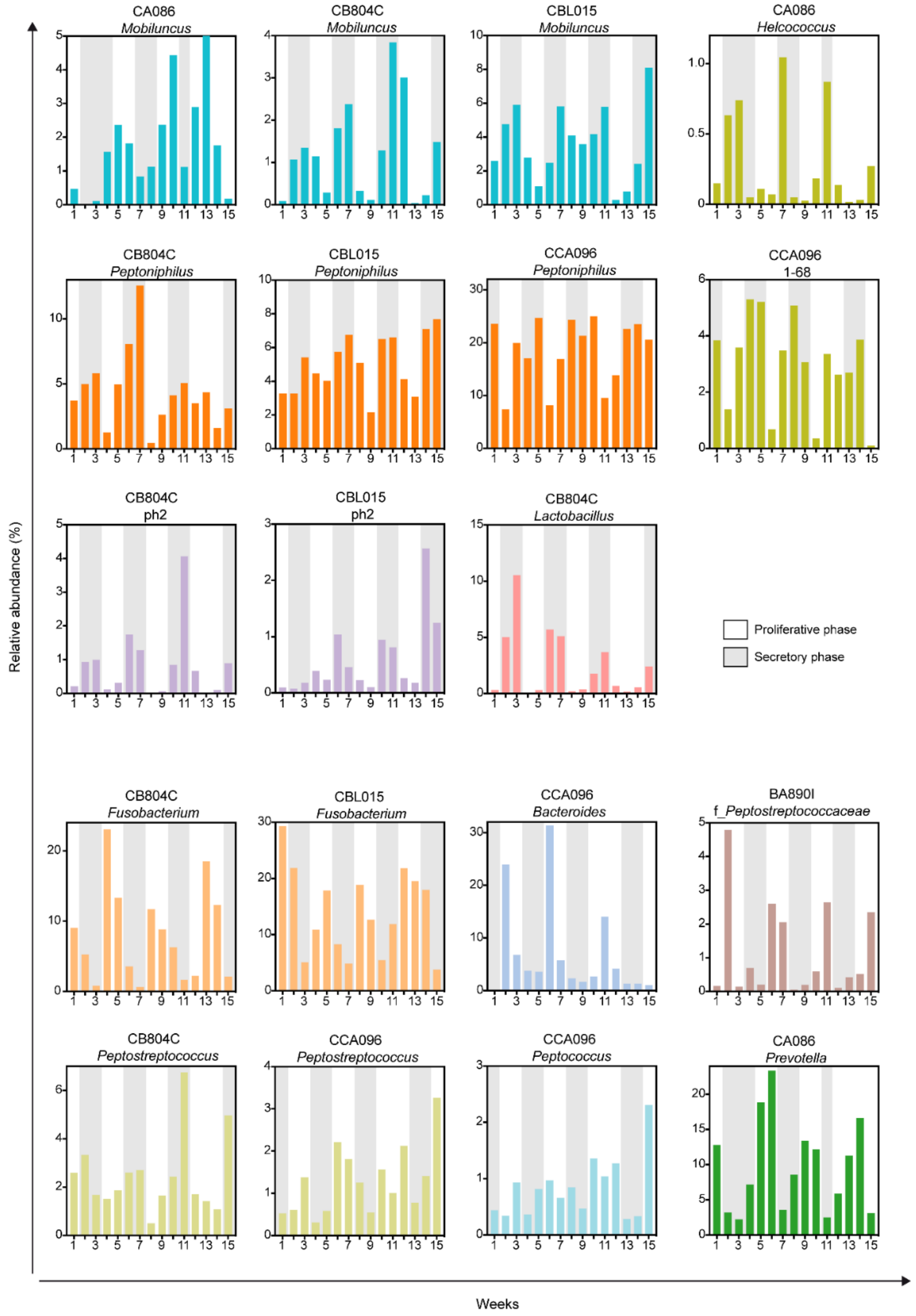

Supplementary Figure 1. Kinetic profiles of vaginal microbial taxa found to be statistically associated with progesterone levels. The relative taxa abundance and progesterone levels are shown for individual animals for 13 of the 15 taxa found to be statistically different between samples taken when progesterone levels were high (≥ 1 ng/ml, grey rectangles) *versus* those taken when they were low level (< 1 ng/ml, white rectangles). The histograms indicate the relative taxa abundance.

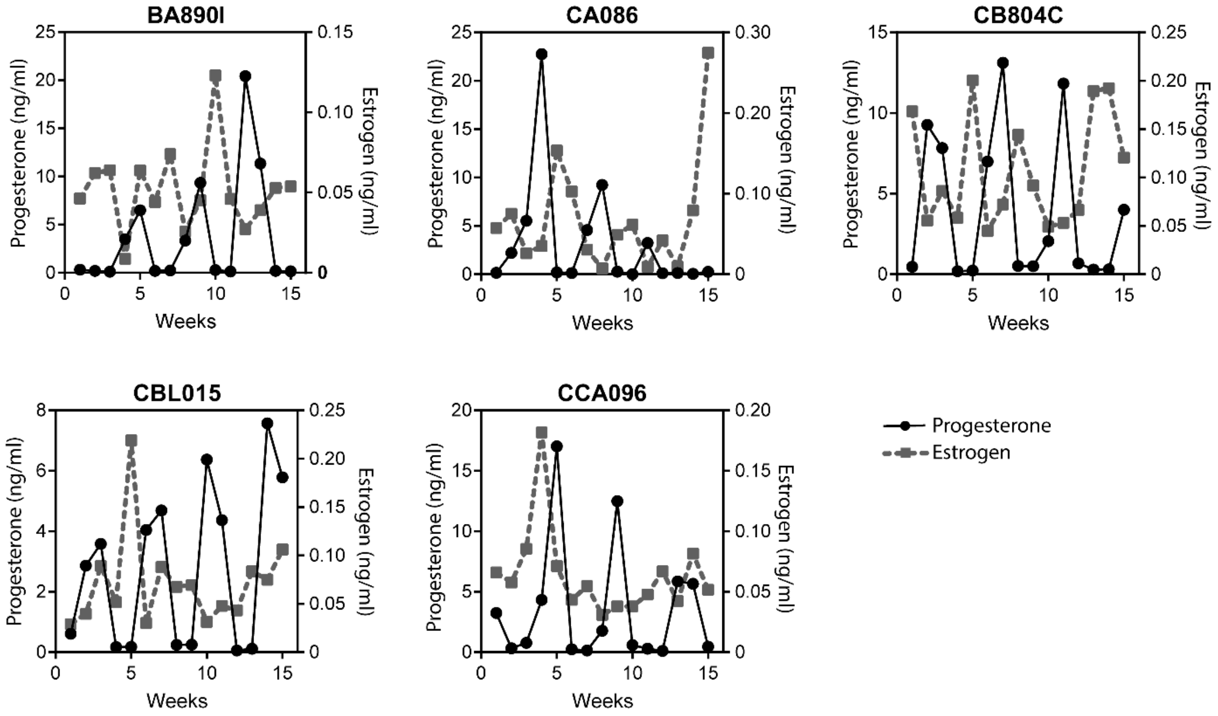

Supplementary Figure 2. Progesterone and estradiol concentrations. The concentrations of progesterone and estradiol were measured each week during the study and are shown for each animal.
